# Regional oncology network between pancreatic centers safeguards waiting times for pancreatoduodenectomy

**DOI:** 10.1007/s13304-019-00677-6

**Published:** 2019-09-10

**Authors:** M. Willemijn Steen, Claire van Vliet, Sebastiaan Festen, Marc G. Besselink, Michael F. Gerhards, Olivier R. Busch, For the Gastrointestinal Oncology Center Amsterdam (GIOCA)

**Affiliations:** 1 Department of Surgery, OLVG, Oosterpark 9, 1091 AC Amsterdam, The Netherlands; 2 Department of Surgery, Cancer Center Amsterdam, Amsterdam UMC, University of Amsterdam, Meibergdreef 9, 1105 AZ Amsterdam, The Netherlands

**Keywords:** Pancreatoduodenectomy, Pancreatic cancer, Pancreatic neoplasms, Waiting lists

## Abstract

Pancreatoduodenectomy (PD) is increasingly performed in high-volume centers, which may compromise waiting times. The aim of this study was to evaluate patient flow and outcome of PD within a regional oncology network of two high-volume centers. A post hoc analysis of a partially retrospective and prospective database was performed of all patients who underwent PD for pancreatic or periampullary neoplasms in both centers of the Gastrointestinal Oncology Center Amsterdam, a collaboration between an academic center and affiliated general teaching hospital, from 2010 to 2014. Outcomes included waiting time to surgery and postoperative morbidity and mortality. A total of 525 PDs were performed, 329 in the academic center (annual volume 66) and 196 in the teaching hospital (annual volume 39). Neoadjuvant treatment was more often used in the academic center, other baseline characteristics were similar. Overall time to surgery was 26 days, which was significantly less in the teaching hospital. The major postoperative morbidity rate was 38.3% (*n* = 201), and the 30- and 90-day mortality was 2.3% and 3.6%. A regional oncology network between an academic center and a general teaching hospital for PD can be an attractive option to safeguard waiting times in selected patients, without compromising outcome.

## Background

Pancreatoduodenectomy (PD) is a low-volume, high-complex procedure with substantial morbidity and mortality. Nationwide centralization of PD in The Netherlands has resulted in reduction of mortality and in high-volume centers also improved overall survival [1–3].

Centralization does pose logistic challenges for high-volume centers. Due to the high demand for operation time and the natural fluctuation in patient numbers, it can be challenging to maintain acceptable waiting times, especially since a Dutch multicenter randomized trial has shown that patients with cholestasis (bilirubin level of 40–250 μmol per liter) should undergo an early PD, rather than preoperative biliary drainage [4].

In 2009, the Gastrointestinal Oncology Center Amsterdam (GIOCA) was founded to improve the logistics for patients with pancreatic and periampullary neoplasms and other gastrointestinal cancers. GIOCA is a cooperation of an academic center [Amsterdam University Medical Center (Amsterdam UMC), location Academic Medical Center] with a general, teaching hospital [Onze Lieve Vrouwe Gasthuis (OLVG)], both situated in Amsterdam but serving a larger catchment area. The main goal of this cooperation was to offer the best, highly specialized multidisciplinary care to all patients, while at the same time shortening the diagnostic process and reducing waiting time to surgery [5]. For this purpose, patient assessment is organized efficiently in 1 day, including imaging, multiple consultations with various specialists and a multidisciplinary meeting.

Some patients seen at the GIOCA outpatient clinic in Amsterdam UMC are operated in the teaching hospital. Hereby, selection takes places as patients with neoadjuvant therapy are operated only in the academic center and patients requiring an urgent PD without preoperative biliary drainage are preferably referred to the teaching hospital.

The aim of this study is to evaluate the outcomes of patients undergoing PD since the introduction of a regional oncology network between an academic center and affiliated teaching hospital, in terms of postoperative morbidity and mortality as well as waiting time for surgery.

## Methods

### Patients and design

A post hoc analysis of partially retrospectively and partially prospectively collected data was performed. All consecutive patients were included who were presented at GIOCA’s outpatient clinic in Amsterdam UMC, location AMC, or at the teaching hospital’s outpatient clinic, and who underwent PD for pancreatic cancer or other periampullary neoplasms between January 1, 2010 and December 31, 2014. This study was approved by the medical ethics review committee of the teaching hospital.

### GIOCA outpatient clinic

Patients are mainly referred from other hospitals and GIOCA therefore functions as a tertiary center. They can also be directly referred by their general practitioner. Patients are seen within 1 week from referral. During a 1 day visit, patients and their relatives are provided with a diagnosis and treatment plan. During this day, the patient may have additional imaging if needed and can meet one or more medical specialists before and after the pancreatobiliary multidisciplinary team meeting. Both hospitals employ their own medical staff, but the multidisciplinary team includes gastroenterologists, radiologists, radiotherapists, pathologists and surgeons of both centers. To minimize waiting times from diagnosis to surgery, GIOCA facilitates shifting care within the academic center and affiliated general, teaching hospital. Surgeons from both centers take turns in running this clinic and personally see the same patients both in the morning and afternoon. Simultaneously, a gastroenterologist and medical oncologist (or residents) see patients in the GIOCA clinic.

Once the indication for PD is determined, the location of surgery is chosen. As the teaching hospital is more flexible in planning surgical cases, patients who are ready for surgery may be referred to the teaching hospital for an early PD, for example, jaundiced patients without indication for biliary drainage or neoadjuvant therapy. Patients initially seen at the teaching hospital were not referred to the GIOCA outpatient clinic and underwent PD at this location.

At both hospitals, surgery is performed by one of their own attending surgeons. Postoperative care and treatment of complications are largely similar between both centers, except for the availability of percutaneous transhepatic cholangiography (PTC) in patients with non-dilated bile ducts. This is performed in the academic center. All other radiological interventions (i.e., abscess drainage and PTC in dilated bile ducts) are also performed in the teaching hospital.

### Definitions

Waiting time is defined as the time in days between the first visit at the outpatient clinic of one of both clinics and the day of surgery. Operative time is defined as the time from incision to final skin closure. Resection margins are classified according to the Royal College of Pathologists Protocol [6]. All complications are classified using the Clavien–Dindo classification [7] with a major complication defined as a Clavien–Dindo score of III or more. Postoperative pancreatic fistula (POPF), delayed gastric emptying (DGE) and postpancreatectomy hemorrhage (PPH) are defined according to the International Study Group on Pancreatic Surgery (ISGPS) definitions [8–10]. Bile leakage (BL) is defined according to the International Study Group on Liver Surgery (ISGLS) definition [11]. Surgical site infection is defined according to the Center for Disease Control and Prevention (CDC) definition [12]. Failure to rescue is defined as death from a major complication (Clavien–Dindo ≥ 3).

### Data collection

Data were partially prospectively and retrospectively collected from patient records. Baseline characteristics included sex, age, body mass index, American Society of Anesthesiologists (ASA) physical status, history of abdominal surgery, diabetes, preoperative biliary drainage, neoadjuvant therapy, and operative and pathological characteristics. Pathological characteristics were presented for pancreatic ductal adenocarcinoma only, as results would otherwise be heterogeneous due to various cancer diagnoses.

Outcomes were waiting time, postoperative complications, reintervention rate, intensive care unit (ICU) admission rate, readmission rate, 30-day and 90-day mortality and failure to rescue.

### Statistical analysis

Data were analyzed using IBM SPSS Statistics for Windows version 22.0 (IBM Corporation, Armonk, NY, USA). Normality of the data was determined by assessment of the histogram. Normally distributed continuous data are presented as means with standard deviations (SD). Non-normally distributed continuous data are presented as medians with interquartile ranges (IQR). Categorical data are presented as numbers with percentages.

Differences in patient characteristics, operation characteristics and pathological characteristics were analyzed by Pearson’s Chi square test for categorical variables and the independent samples *t* test or Mann–Whitney *U* test according to the distribution for continuous variables. Two-tailed *p* values < 0.05 were considered statistically significant. A multivariable logistic model was used to examine differences in the following outcomes between the academic center and teaching hospital: postoperative complications, reinterventions, ICU admission, readmission, 30-day and 90-day mortality and failure to rescue. Variables included in these models were ASA classification, neoadjuvant therapy, waiting time, surgical technique, operative time and radicality. Although the amount of blood loss was different between the hospitals, no correction was made for blood loss due to collinearity with the outcome measures. Data are presented as odds ratio (OR) with 95% confidence interval (CI). Bonferroni correction was applied for multiple testing. As 15 outcomes were tested, a *p* value < 0.003 was considered statistically significant (0.05/15).

## Results

Overall, 525 patients who had undergone a PD were included: 473 patients (90.1%) were seen at the GIOCA outpatient clinic and 52 patients (9.9%) directly at the teaching hospital outpatient clinic. In total, 329 patients (62.7%) underwent PD in the academic center and 196 (37.7%) in the teaching hospital. Of all 196 patients who underwent PD in the teaching hospital, 144 (73.5%) were referred from the GIOCA outpatient clinic and 52 patients (26.5%) were seen at the teaching hospital’s outpatient clinic. Patients who underwent PD in the academic center were all seen at the GIOCA outpatient clinic.

### Patient operative and pathological characteristics

Patient characteristics are presented in Table 1. Overall median waiting time was 26 days. The waiting time was shorter in the teaching hospital (median 14 versus 33 days; *p* < 0.01), also of patients without neoadjuvant therapy (median 14 versus 32 days; *p *< 0.01).

**Table 1:** Baseline characteristics of patients undergoing PD for pancreatic or periampullary neoplasms in two hospitals within the GIOCA network

	Total population (*n* = 525)	Teaching hospital (*n* = 196)	Academic center (*n* = 329)	*p* value
Male sex	289 (55.0)	110 (56.1)	179 (54.4)	0.70
Age [years; mean (SD)]	64 (11)	64 (11)	64 (12)	0.55
BMI [kg/m^2^; mean (SD)]	25 (5)	25 (5)	25 (5)	0.91
ASA physical status				0.05
ASA 1	97 (18.5)	40 (20.4)	57 (17.4)	
ASA 2	333 (63.4)	112 (57.1)	221 (67.4)	
ASA 3	94 (17.9)	44 (22.4)	50 (15.2)	
Previous abdominal surgery	169 (32.2)	62 (31.6)	107 (32.5)	0.83
Diabetes	115 (21.9)	39 (19.9)	76 (23.2)	0.38
Preoperative biliary drainage	251 (47.8)	85 (43.8)	166 (50.8)	0.13
Neoadjuvant therapy	23 (4.4)	–	23 (7.0)	< 0.01

Data are presented as *n* (%) unless otherwise specified

*ASA* American Society of Anesthesiologists, *BMI* body mass index, *GIOCA* Gastrointestinal Oncology Center Amsterdam, *IQR* interquartile range, *PD* pancreatoduodenectomy, *SD* standard deviation

Operative and pathological characteristics are presented in Table 2. In the teaching hospital a few more laparoscopic procedures were performed, operation time was shorter, blood loss was less and R0 rate was higher.

**Table 2:** Operative and pathological characteristics of patients undergoing PD for periampullary neoplasms in two hospitals within the GIOCA regional oncology network

	Total population (*n* = 525)	Teaching hospital (*n* = 196)	Academic center (*n* = 329)	*p* value
Surgical technique				0.02
Open	512 (97.5)	187 (95.4)	325 (98.8)	
Laparoscopy	13 (2.5)	9 (4.6)	4 (1.2)	
Type of resection				0.08
Classic Whipple	125 (23.8)	55 (28.1)	70 (21.3)	
Pylorus preserving PD	400 (76.2)	141 (71.9)	259 (78.7)	
Venous resection	79 (15.0)	30 (15.5)	49 (15.0)	0.88
Operation time [min; mean (SD)]	297 (99)	283 (79)	305 (108)	0.01
Blood loss [mL; median (IQR)]	500 (300-1000)	400 (300-753)	645 (350-1100)	< 0.01
Pathological diagnosis				0.08
Pancreatic ductal adenocarcinoma	205 (39.0)	91 (46.4)	114 (34.7)	
Distal cholangiocarcinoma	92 (17.5)	37 (18.9)	55 (16.7)	
Ampulla of Vater carcinoma	79 (15.0)	26 (13.3)	53 (16.1)	
IPMN	31 (5.9)	5 (2.6)	26 (7.9)	
Duodenal carcinoma	32 (6.1)	11 (5.6)	21 (6.4)	
Neuroendocrine tumor	19 (3.6)	3 (1.5)	16 (4.9)	
Pseudopapillary tumor	3 (0.6)	1 (0.5)	2 (0.6)	
Chronic pancreatitis	27 (5.1)	11 (5.6)	16 (4.9)	
Other benign conditions	20 (3.8)	6 (3.1)	14 (4.3)	
Other malignant neoplasms	16 (3.0)	5 (2.6)	11 (3.3)	
Tumor size [cm; mean (SD)]	2.9 (1.8)	2.7 (1.2)	3.1 (2.1)	0.06
Tumor stage^a^				0.10
T1	15 (7.7)	10 (11.1)	5 (4.8)	
T2	32 (16.4)	18 (20.0)	14 (13.3)	
T3	140 (71.8)	57 (63.3)	83 (79.0)	
T4	8 (4.1)	5 (5.6)	3 (2.9)	
Lymph node stage^a^				0.65
N0	51 (25.1)	24 (26.7)	27 (23.9)	
N1	152 (74.9)	66 (73.3)	86 (76.1)	
Radicality^a^				
R0	114 (55.3)	62 (67.4)	52 (45.6)	< 0.01
R1	83 (40.3)	22 (23.9)	61 (53.5)	
R2	9 (4.4)	8 (8.7)	1 (0.9)	

Data are presented as *n* (%) unless otherwise specified

*ASA* American Society of Anesthesiologists, *GIOCA* Gastrointestinal Oncology Center Amsterdam, *IPMN* intraductal papillary mucinous neoplasm, *IQR* interquartile range, *PD* pancreatoduodenectomy, *SD* standard deviation

^a^Data presented for patients with pancreatic ductal adenocarcinoma only

### Postoperative morbidity and mortality

The overall major morbidity rate was 38.3% (*n* = 201). The median length of hospital stay was 12 days. Overall 30-day mortality was 2.3% (*n* = 12) and overall 90-day mortality was 3.6% (*n* = 19).

When comparing postoperative morbidity and mortality between both centers, outcomes were adjusted for possible confounders. Outcomes are presented in Table 3. Postoperative 30-day and 90-day mortality were non-significantly higher in the teaching hospital [teaching hospital versus academic center: 30-day mortality 3.9% (*n* = 7) versus 1.6% (*n* = 5); 90-day mortality 6.3% (*n* = 11) versus 2.6% (*n* = 8)]. Furthermore there were no differences in length of stay at the ICU (academic center versus teaching hospital − 1.14 days; 95% CI − 5.59 to 3.31; *p *= 0.61) and the total length of hospital stay (academic center versus teaching hospital − 0.70 days; 95% CI − 3.36 to 19.96; *p *= 0.60).

**Table 3:** Postoperative morbidity and mortality after PD in two hospitals within the GIOCA network (teaching hospital versus the academic center), adjusted for confounders (ASA classification, neoadjuvant therapy, waiting time, surgical technique, operative time and radicality)

	OR (95% CI)	*p* value*
Major complication Clavien–Dindo ≥ III	0.90 (0.58–1.39)	0.621
Postoperative pancreatic fistula grade B/C	1.14 (0.67–1.92)	0.633
Delayed gastric emptying grade B/C	0.47 (0.28–0.79)	0.004
Postpancreatectomy hemorrhage grade B/C	0.60 (0.28–1.28)	0.186
Bile leakage grade B/C	1.50 (0.71–3.14)	0.287
Chyle leakage	1.09 (0.63–1.90)	0.755
Surgical site infection	1.72 (0.92–3.22)	0.090
Reintervention	0.89 (0.57–1.39)	0.615
ICU admission	0.61 (0.33–1.12)	0.110
Readmission within 30 days	1.25 (0.70–2.24)	0.448
30-day mortality	4.15 (0.90–19.08)	0.067
90-day mortality	2.96 (0.96–9.15)	0.059
Failure to rescue	1.95 (0.51–7.53)	0.332

*GIOCA* Gastrointestinal Oncology Center Amsterdam, *ICU* intensive care unit, *IQR* interquartile range, *PD* pancreatoduodenectomy

*A *p* value < 0.003 is considered statistically significant, as Bonferroni correction is applied for multiple testing

## Discussion

Our study demonstrates that a regional oncology network between an academic center and a teaching hospital for the treatment of pancreatic and periampullary neoplasms can give good outcomes in combination with short waiting times.

With the aim of improving health care for patients with periampullary neoplasms, centralization of PD has taken place in The Netherlands during the past decades. Centralizing PD has resulted in reduced nationwide mortality. The in-hospital mortality decreased from 9.8% in 2010 to 5.1% in 2015 [1] and a recent study demonstrated further improvement of the 90-day mortality of 4.3% in centers performing > 40 PDs annually [2]. Overall survival was also found to be superior in high-volume centers (> 20 PDs annually) compared to low-volume centers (< 20 PDs annually), with a median survival of 18 versus 16 months [1]. It has also resulted in increased resection rates from 10.7% in 2000–2004 to 15.3% in 2005–2009 [3]. In 2011, the Dutch Society for Surgery set a minimum volume of 20 PDs annually and a mandatory nationwide audit (Dutch Pancreatic Cancer Audit) was installed in 2013 by the Dutch Pancreatic Cancer Group [13].

With the ongoing increase of PD volume in high-volume centers, the pressure on operating room capacity is rising, with the danger of concomitant increase in waiting times. In The Netherlands, the national guideline for pancreatic cancer recommends pancreatic resection within 3 weeks from the multidisciplinary meeting [14]. This does not apply to a selected group of patients who participate in research on neoadjuvant treatment strategies, or to patients with an indication for preoperative biliary drainage. Overall, patients with jaundice (bilirubin level of 40–250 μmol per liter) should undergo early surgery without preoperative biliary drainage if neoadjuvant treatment is not indicated [4]. The impact of the time interval from diagnosis to surgery on oncologic outcomes of periampullary neoplasms, such as resection rate and survival, has not yet been clarified [15–18]. However, waiting time has revealed to have a negative impact on the quality of life and is one of the quality indicators for care [19–21]. Therefore, it remains advisable to strive for a short waiting time, once the indication for surgery is set and the patient is ready to undergo the operation.

Centralization of PD to high-volume centers collaborating within oncology networks might bring a solution as long as high-quality care is guaranteed. Within the described GIOCA collaboration, the 30-day and 90-day mortality is within the range of other high-volume centers, which has been reported to be 0–3.8% versus 4.3–7.3%, respectively [2, 22–25].

The collaboration offers several advantages. The academic center provides state-of-the-art diagnostic workup in 1 day and selects patients for the best treatment and location. The teaching hospital is able to maintain short waiting times due to its operation flexibility to shift with its capacity, whereas the academic center focuses on complex and time-consuming procedures. By this cooperation, selected patients are able to benefit from short waiting times, especially patients in whom preoperative biliary drainage can be prevented by early surgery, and also other patients who are directly ready for surgery such as patients who do not require other preoperative (cardiologic) consultations or neoadjuvant treatment. By further intensification of this collaboration, waiting in the academic hospital may be improved until the target value of 3 weeks is achieved.

Although not statistically significant, one might argue that a possible trend may be seen toward a worse 30-day and 90-day mortality in the teaching hospital compared to the academic center. However, we do believe that patients treated in the teaching hospital also benefit from the regional oncology network in reducing the risk of ‘failure to rescue’. For example, patients with postoperative bile leakage who were operated in the teaching hospital may be referred for percutaneous transhepatic cholangiography of non-dilated bile ducts to the academic center, where there is extensive experience with this technique. Postoperative outcomes may be further improved by reinforcement of the collaboration: by sharing postoperative care, but also by better patient selection for referral. Within our data with low mortality, it was unfortunately not possible to perform a multivariate analysis for 30-day and 90-day mortality, due to the small sample size. In the literature several predictors for mortality have been demonstrated such as age and extensive comorbidity [26–31], and in the future these criteria may be used for further selection of patients for referral.

As patients were selected for referral to the teaching hospital, there were some differences between both centers in surgical characteristics, reflecting the decision tree for referral. Patients with neoadjuvant therapy were all operated in the academic center, as neoadjuvant treatment is not available in the teaching hospital. Furthermore, there was a trend toward more preoperative biliary drainage in the academic center. This is due to the selection of patients with obstructive jaundice for referral to the teaching hospital for PD, due to a shorter waiting list and therefore to prevent them from undergoing preoperative biliary drainage. No other preset criteria for patient referral were applied, for example there was a comparable number of vascular resections in both centers. Also, the finding of differences in surgical characteristics (shorter operating time, less blood loss and more R0 resections in the teaching hospital) suggests that less complex patients underwent PD in the teaching hospital as a result from selection in patient referral. Unfortunately, our data do not provide more insight into the general health of patients, except for ASA classification, like cardiac comorbidity. Thus, other reasons, than logistic reasons, for a longer time interval to treatment in the academic center, for example a preoperative cardiac workup for surgery, have not been addressed.

There were also differences between both centers in pathological characteristics. There was a tendency toward larger and more advanced (T3) tumors in patients treated in the academic center. Another difference in pathological characteristics was the radical resection rate. There were more R0 resections and less R1 resections in the teaching hospital compared to the academic center. This could be related to the presence of dedicated pancreatic pathologists in the academic center, but this is speculative. It is known that when more experienced pathologists judge pancreatic specimens, the R1 rate increases [28]. Therefore, we intend to expand our collaboration also to other specialties such as pathologists, gastroenterologists, medical oncologists and radiologists. Differences in pathological outcomes could also be a result from patient selection, with less complex patients operated in the teaching hospital.

This study does have several limitations. First, the retrospective study design might have introduced bias. By default, therefore, groups differed in baseline characteristics and operative and pathological characteristics, which may partially be explained by patient selection, before referral. Second, the teaching hospital also operated on patients who were not seen in the GIOCA clinic. We are currently addressing this point to explore the possibility to see also all patients from the teaching hospitals in the GIOCA clinic.

## Conclusion

A regional oncology network between two different hospitals with different faculties (an academic center and a teaching hospital) with patient selection for PD can improve the access to tertiary care with short waiting times without compromising the outcomes of care.
